# Various aspects of inflammation in heart failure

**DOI:** 10.1007/s10741-019-09875-1

**Published:** 2019-11-09

**Authors:** Mieczysław Dutka, Rafał Bobiński, Izabela Ulman-Włodarz, Maciej Hajduga, Jan Bujok, Celina Pająk, Michał Ćwiertnia

**Affiliations:** 1 Faculty of Health Sciences, Department of Biochemistry and Molecular Biology, University of Bielsko-Biala, Willowa St. 2, 43-309 Bielsko-Biala, Poland; 2 Faculty of Health Sciences, Department of Emergency Medicine, University of Bielsko-Biala, Willowa St. 2, 43-309 Bielsko-Biala, Poland

**Keywords:** Heart failure, Left ventricular remodelling, Inflammation, Biomarkers, Micro-RNA

## Abstract

Despite significant advances in the prevention and treatment of heart failure (HF), the prognosis in patients who have been hospitalised on at least one occasion due to exacerbation of HF is still poor. Therefore, a better understanding of the underlying pathophysiological mechanisms of HF is crucial in order to achieve better results in the treatment of this clinical syndrome. One of the areas that, for years, has aroused the interest of researchers is the activation of the immune system and the elevated levels of biomarkers of inflammation in patients with both ischaemic and non-ischaemic HF. Additionally, it is intriguing that the level of circulating pro-inflammatory biomarkers correlates with the severity of the disease and prognosis in this group of patients. Unfortunately, clinical trials aimed at assessing interventions to modulate the inflammatory response in HF have been disappointing, and the modulation of the inflammatory response has had either no effect or even a negative effect on the HF prognosis. The article presents a summary of current knowledge on the role of immune system activation and inflammation in the pathogenesis of HF. Understanding the immunological mechanisms pathogenetically associated with left ventricular remodelling and progression of HF may open up new therapeutic possibilities for HF.

## Introduction

Heart failure (HF) is a clinical syndrome typically characterised by the appearance of symptoms such as dyspnoea, a worsening tolerance to exercise, which may be accompanied by abnormalities in a physical examination (e.g. features of pulmonary stasis, peripheral oedema). These result, in HF, from abnormalities in the structure and/or function of the heart, leading to insufficient blood supply to the tissue [1]. This definition applies only to symptomatic patients. However, it should be remembered that many patients have asymptomatic dysfunction of the left ventricle long before the first diagnosis of HF. However, due to the lack of symptoms, they are not diagnosed and are not treated earlier. Depending on the type of structural and/or functional disorder of the heart, three categories of HF are currently distinguished: HF with reduced left ventricle ejection fraction (HFrEF), HF with preserved left ventricle ejection fraction (HFpEF) and HF with a mid-range left ventricle ejection fraction (HFmrEF) [1]. Therefore, in order to diagnose HF, the coexistence of clinical symptoms and abnormalities in the structure and/or function of the heart is now necessary. These abnormalities lead either to decreased ejection volume of the heart or to elevated left ventricular filling pressure with cardiac output maintained.

In addition, according to the timeline and the dynamics of the appearance of symptoms, either chronic HF (CHF) or acute HF may be diagnosed. The causes of HF can be divided into the following: (1) associated with myocardial disease (ischaemic heart disease, toxic damage, inflammation-related and immune-related damage—infectious and non-infectious, infiltrative diseases, metabolic disorders and genetic syndromes), (2) associated with abnormal preload/afterload of the heart (hypertension, valvular heart diseases, pericardial syndromes and endocarditis), (3) associated with arrhythmias and conduction disorders (tachyarrhythmia and bradyarrhythmia) [1].

Regardless of the aetiology, significant neurohormonal activation emerges in HF which plays an important role in the pathophysiology of HF. Therefore, biomarkers of this neurohormonal activation, such as the B-type natriuretic peptide (BNP) and its biologically inactive N-terminal fragment (NT-proBNP), are now widely used in clinical practice. They have both diagnostic and prognostic value in HF. As the basic processes underlying structural and functional abnormalities in HF are progressive fibrosis and heart remodelling, the processes that stimulate these disorders have been the subject of numerous studies. The most important of these include inflammation and activation of the immune system, which, it has been confirmed, significantly stimulate cardiac fibrosis and remodelling and therefore contribute to the progression of HF. So far, a lot of experimental evidence has been gathered confirming the participation of inflammation in the development and course of different types of HF [2–6]. Several inflammatory biomarkers have also been evaluated, assessing their usefulness as diagnostic and prognostic indicators in HF [2, 5, 6]. In addition, various anti-inflammatory therapeutic strategies in HF have also been assessed, which, unfortunately, most often have not met the hopes placed in them [2, 7, 8]. Some of the aspects of inflammation in HF examined so far are presented in the following subsections of this paper.

## Classic pro-inflammatory cytokines and monocytes in HF

C-reactive protein (CRP) is considered a classic marker of inflammation. The plasma concentration of CRP is elevated in patients with HF and is considered an independent prognostic indicator of future adverse events in this group of patients [9–14]. CRP stimulates monocytes to produce pro-inflammatory cytokines [9]. Its usefulness as a prognostic indicator in HF has been studied in, among others, patients with HFpEF isolated from the LURIC (Ludwigshafen Risk and Cardiovascular Health) patient population [15]. From the population of this study, 506 patients were identified as meeting the diagnostic criteria of HFpHF, and, after excluding acute or chronic infection, autoimmune disease and cancer, 459 patients were qualified for the study. This study showed that plasma CRP levels were significantly, positively correlated with clinical and laboratory HF severity indices, such as the New York Heart Association (NYHA) and NT-proBNP. In addition, CRP proved to be a strong and independent predictor of total mortality and a particularly strong predictor of cardiovascular mortality [15]. Interestingly, CRP turned out to be a stronger predictive indicator in HFpEF in a subset of patients without coronary artery disease (CAD) than in CAD patients. This is interesting mainly due to the known contribution of CRP in the induction of the expression of adhesion molecules on the vascular endothelium and of the migration and transformation of monocytes and, consequently, in the induction and progression of the atherosclerotic process. However, what is not fully explained is whether CRP is directly involved in the atherosclerotic process or whether it is just a non-specific marker of the ongoing process of immune activation [16, 17]. The result of this study, confirming a stronger predictive value of CRP in HFpEF in patients without CAD may indicate that in HFpEF, immune-induced cardiac abnormalities are more important than atherosclerotic lesions in coronary arteries [15].

The significance of plasma CRP concentration as a marker of HFpEF severity and the degree of burden of significant accompanying diseases in patients with HFpEF has been confirmed in other studies [18–20]. Plasma concentrations of CRP in patients with HFpEF positively correlated with NT-proBNP, the prevalence of chronic obstructive pulmonary disease (COPD), endothelin-1 concentration, aldosterone concentration, body mass index (BMI) and the overall number of comorbidities. Higher plasma CRP concentration was also associated with a higher rate of atrial fibrillation and more frequent right ventricular dysfunction in this patient population [18].

It is well known that the immune system is activated at an early stage of ischaemia and myocardial necrosis during myocardial infarction (MI). The activation of the immune system is perceived as the initiator of repair processes within the myocardial infarct damage area [21]. It has been emphasised that early infiltration by a large number of inflammatory cells, mainly neutrophils and monocytes/macrophages, into the area of MI is particularly important [9, 21]. This cellular immune response and the subsequent inflammatory response were considered to be the primary factor promoting adverse post-infarction remodelling of the left ventricle. Monocytes are a highly diverse group of cells, and the large variation of surface markers allows the identification of many monocyte subtypes with various functions, e.g. monocytes defined as CD14+ CD16+, CD14+CD16− and others are distinguished on the basis of surface markers [9, 22]. Individual subpopulations of monocytes may, under certain conditions, produce pro-inflammatory cytokines, while other subpopulations may produce predominantly anti-inflammatory cytokines [9].

**Fig. 1:**
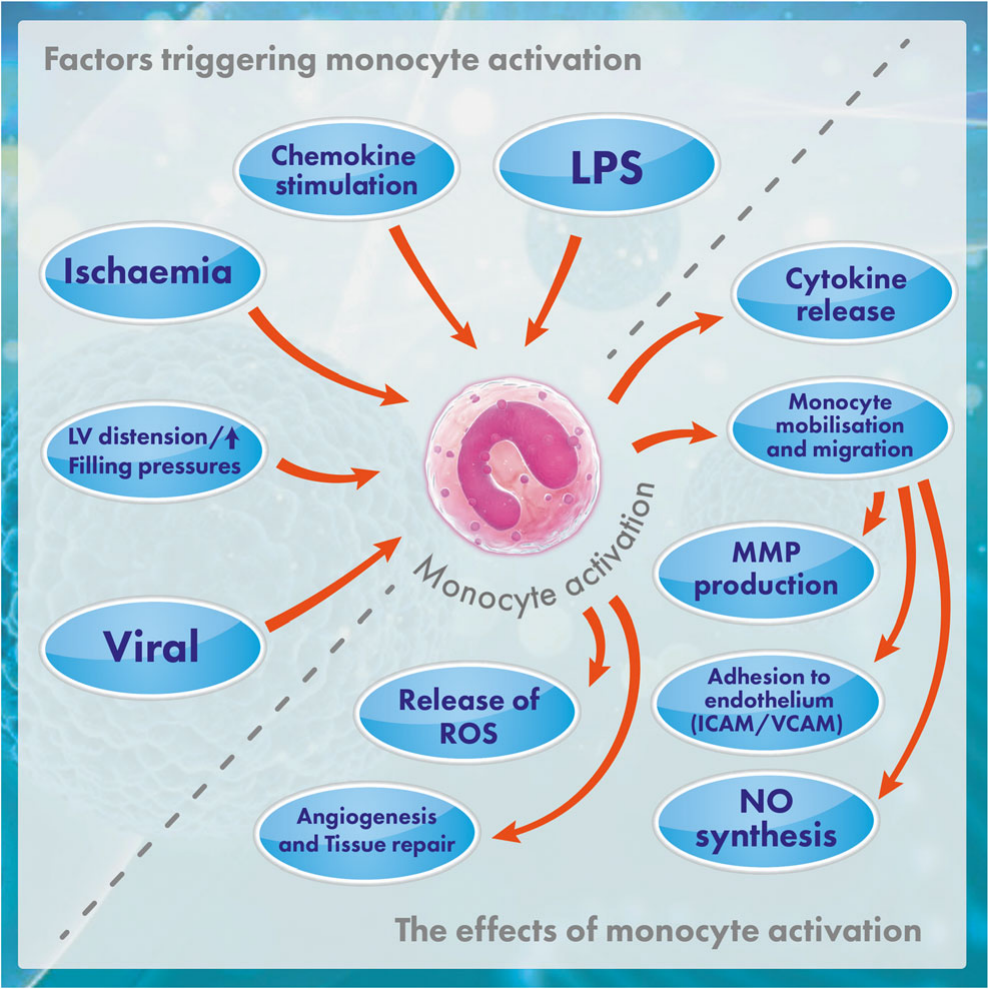
Monocyte activation in HF. This figure shows the basic factors stimulating the activation of monocytes in HF and the basic effects of this monocyte activation. Explanation of abbreviations: LPS lipopolysaccharide, MMP matrix metalloproteinases, NO nitric oxide, ICAM intercellular adhesion molecule, VCAM vascular cell adhesion molecule, ROS reactive oxygen species

Infiltration of the damaged myocardium by monocytes is not limited to the most frequent, ischaemic type of damage to the myocardium. When the damage to the myocardium is for other reasons, such as infection, left ventricular pressure overload or primary muscular pathology, this also gives rise to the infiltration of the myocardium by the monocytes and to their activation. The evidence for the activation of monocytes in HF includes the increase in the plasma concentration of neopterin, which is a specific marker of monocyte activation [11, 23]. The plasma concentration of neopterin in patients with HF correlates with the concentration of tumour necrosis factor-alpha (TNF-alpha). The mechanism of monocyte activation in HF, however, is extremely complex [9] (Fig. 1). Recently, it has also been highlighted that certain subpopulations of activated macrophages, termed M2 or CD206+F4/80+CD11b+, infiltrating the area of MI, show remedial abilities in the area of ischaemic heart damage [21].

Elevated plasma TNF-alpha concentration in patients with HF is a well-known fact. Plasma concentrations of TNF-alpha correspond well with the functional classification according to NYHA of patients with HF and with plasma concentrations of classic HF biomarkers, such as NT-proBNP [9, 10, 24]. A high content of TNF-alpha was also found in failing hearts collected during transplantation [9]. It has been shown in animal studies that TNF-alpha is biologically active thanks to its binding with two different receptors—TNF receptor 1 (TNFR 1) and TNF receptor 2 (TNFR 2). In an experimental model of MI in TNFR 1 knockout mice, it was demonstrated that the lack of this receptor influenced the improvement of left ventricular contractility after MI. In contrast, the induction of experimental MI in TNFR 2 knockout mice resulted in the opposite effect, i.e. the intensification of ventricular dilatation and dysfunction [25, 26].

In clinical trials, in a group of patients with HF, it was confirmed that TNFR 2 plasma levels were significantly associated with the degree of diastolic dysfunction in patients with HFpEF but not HFrEF. At the same time, it has been confirmed that plasma levels of both TNFR 1 and TNFR 2 were significantly associated with the severity of symptoms in both types of HF [10, 27]. It is believed that TNFR 2 high plasma concentrations may reflect the loss of protective signalling mechanisms in the tissue that removes TNFR 2, leading to its increased plasma concentrations. In this way, the correlation between TNFR 2 plasma levels and both the severity of diastolic dysfunction and the severity of symptoms in patients with HFpEF can be explained. It is also suggested that TNFR 2 plasma concentration may be a good biomarker of the level of severity previously diagnosed by HFpEF [27].

In HF, TNF-alpha also stimulates the production by leukocytes of neutrophil gelatinase-associated lipocalin (NGAL), whose high plasma concentrations in HF patients are considered to be a strong prognostic indicator associated with higher mortality in HF [28]. TNF-alpha leads to increased production of NGAL by leukocytes in HF through the stimulation of TNFR 1. Interestingly, elevated plasma levels of NGAL in patients with HF transpired to be a specific marker for somatic symptoms of depression in this group of patients [28]. NGAL is strongly pathophysiologically associated with the inflammatory process underlying HF, and, at the same time, its plasma concentrations are significantly elevated in HF patients with coexisting somatic symptoms of depression. Therefore, this parameter can be considered as an important biological element responsible for adverse prognosis in HF patients with accompanying somatic symptoms of depression [28].

Interesting data was also provided by studies in which the participation of CD4+ T cells in repair processes and positive left ventricular remodelling after MI was confirmed during experimental MI in animals [29]. During the inflammatory phase of post-infarction repair, cardiac fibroblasts (CFs), activated by interleukin-1 (IL-1), acquire a pro-inflammatory phenotype and secrete cytokines and chemokines. Such pro-inflammatory activation of CFs inhibits alpha-smooth muscle actin and delays myofibroblast conversion. During the next proliferative phase of post-infarction repair, fibroblasts may transform into myofibroblasts, and further subsets of reparative fibroblasts are recruited and activated, which is important in the scar formation process [21].

Also, in clinical trials, the correlation between the degree of left ventricular dysfunction in patients with HF and both the circulating inflammation cells and the biomarkers of inflammation was confirmed [21]. Increased plasma concentrations of biomarkers such as TNF-alpha, ST2, IL-1, interleukin-6 (IL-6), interleukin-8 (IL-8), Gal-3 (Gal-3) or growth differentiation factor 15 (GDF15) are considered as characteristic for HF [2, 19, 21]. IL-1 acts on virtually all cells of the immune system, including neutrophils, macrophages, eosinophils and mast cells. ST2 is a cytokine which belongs to the cytokine IL-1 superfamily and acts as a receptor for interleukin-33 (IL-33). IL-33 is secreted by myocytes in response to their mechanical stretching and is considered as a marker of inflammation [30]. Gal-3 is released by macrophages in response to tissue damage. It is involved in the activation of fibroblasts, thereby mediating the process of tissue fibrosis. Gal-3 is considered as a marker of fibrosis, and especially in patients with HFpEF, high plasma concentrations of Gal-3 have been shown to be associated with an unfavourable prognosis in this group of patients [23, 31]. It should be emphasised that recently the Food and Drug Administration (FDA) approved these two above-mentioned inflammation biomarkers (ST2 and Gal-3) as prognostic indicators in HF [2]. Another pro-inflammatory cytokine, whose increase in plasma concentration was confirmed in HF, is IL-6, whose role in HF progression is complex. IL-6 has a stimulating effect on the differentiation of B and T lymphocytes and the activation of thymocytes, macrophages and natural killers (NK). IL-6 also stimulates hepatocytes to produce CRP [12]. It was confirmed that, on the one hand, IL-6 may cause myocardial hypertrophy and left ventricular systolic dysfunction, and on the other hand, it may inhibit apoptosis of cardiomyocytes [9]. However, its plasma concentrations, like other pro-inflammatory cytokines such as TNF-alpha or IL-8, correlated with a worse prognosis in the group of patients with HF [9, 21, 32]. IL-8 production is increased through the activation of the NF-kappaB pathway by, among other things, ischaemia. Increased IL-8 expression in the myocardium during acute MI has been confirmed, as has been the value of IL-8 as a predictor of the development of HF after MI [33, 34]. With regard to the GDF15 mentioned above, this belongs to the cytokine TGF-beta superfamily and its expression is particularly high in the inflammation process. Plasma concentrations of GDF15 are significantly elevated in HF and, in clinical trials, the prognostic value of this parameter was confirmed in both the HFrEF and HFpEF patients [35]. GDF15, which is a marker of systemic inflammation, has proved to be an additional prognostic factor in HF, independent of NT-proBNP and highly sensitive troponin T (hsTnT) [35]. This highlights the importance of inflammation in the development of HF.

Yosuke Kayama and colleagues have demonstrated in an animal model that over-expression of cardiac 12/15-lipoxygenase (12/15-LOX) induces inflammation and thus leads to left ventricular systolic dysfunction and HF. They found that the increased expression of this enzyme up-regulates monocyte chemoattractant protein 1 (MCP-1) and, in this way, triggers the infiltration of the heart by macrophages, leading to cardiac fibrosis and left ventricular systolic dysfunction [36]. In addition, it has been confirmed that blocking the activity of MCP-1 in vivo in transgenic Alox 15 mice, in which systolic dysfunction was induced by chronic pressure overload, reduces myocardial infiltration by macrophages, as well as inflammation and fibrosis within the myocardium and, thereby, ultimately reduces the degree of left ventricular systolic dysfunction. [36].

## Toll-like receptors and inflammation in HF

**Fig. 2:**
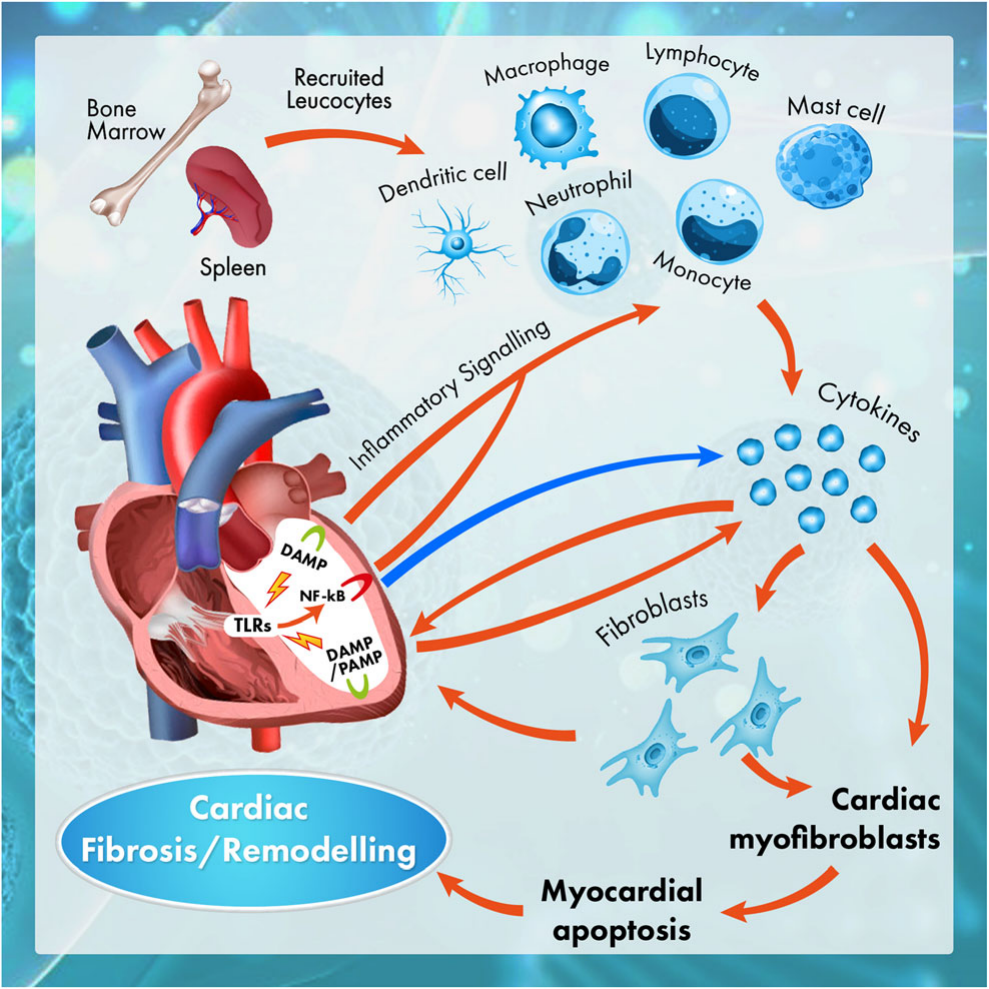
The activation and maintenance of inflammation in HF. This figure shows how the two basic patterns of damage to the myocardium (DAMP and PAMP) lead to the activation and maintenance of inflammation within the myocardium, which ultimately leads to HF. Activation by DAMP and/or PAMP TLRs (these are mainly TLR2, TLR3 and TLR4 which are found in abundance in the myocardium) leads to the activation of NF-kB, which is the basic inflammatory activating factor for inflammation. During myocardial ischaemia, NF-kB is a signalling factor for the production of pro-inflammatory cytokines for many types of cells, including those monocytes infiltrating the myocardium. However, later, the cardiomyocytes themselves, thanks to the increased expression of TLR4 present on their surface, act as pro-inflammatory cells in hearts affected by post-infarction damage. Explanation of abbreviations in the main text

More and more experimental data confirms that in the activation and maintenance of inflammation in HF the key role is played by an interesting family of pattern recognition receptors (PRRs), which includes toll-like receptors (TLRs) [37–41]. These receptors elicit an innate immune response. They are typically activated by both pathogen-associated molecular patterns (PAMPs) and damage-associated molecular patterns (DAMPs). In the first case, the activation results from the action of the pathogenic microorganism and in the second case from the damage to the cells present in the heart. So far, ten types of TLRs have been identified in humans. TLRs 1, 2, 4, 5 and 6 are found on the surface of cells, whereas TLRs 3, 7, 8 and 9 are present in intracellular structures. From the types of TLRs mentioned, mainly TLR2, TLR3 and TLR4 are found in cardiomyocytes. Activation of these receptors leads to the activation of nuclear factor-kappaB (NF-kB), which is the basic transcription factor which activates inflammation [40, 41]. It should be emphasised, however, that the role of NF-kB can differ, depending on whether its activation is short-lived and transient or prolonged [42–44]. In the former case, activation of NF-kB may offer cardioprotection. Where there is prolonged activation of NF-kB, however, this causes both the release of a large amount of pro-inflammatory cytokines and chemokines and an intensification of cardiomyocyte apoptosis [39, 42, 45, 46] (Fig. 2).

Of particular interest is the increased expression of TLR4 on human cardiomyocytes under ischaemic conditions, as confirmed in cell cultures as well as in hearts affected by infarction. Initially, increased TLR4 expression was demonstrated on cardiomyocytes in the acute phase of MI and in the first days after MI [37, 41, 47]. More recently, an animal model of HF induced by MI has demonstrated that the expression of TLR4 on cardiomyocytes also persists 4 weeks after MI, which determines high levels of pro-inflammatory cytokines in both the infarcted area and distal areas of the heart. In this animal model of MI, it has also been demonstrated that injection of lentivirus short hairpin RNA (shRNA) against TLR4 into the infarcted heart significantly reduces the production of pro-inflammatory cytokines, reduces the size of MI and improves heart function [37]. It has been confirmed that infarction-induced inflammation and infiltrates consisting of monocytes/macrophages distant from the infarction zone of the heart area persist 4 to 7 weeks after MI. However, the participation of monocytic/macrophage infiltration in the production of pro-inflammatory cytokines and in the prolongation of inflammation in the areas of the heart distant from the infarct zone does not exceed 2 weeks. More and more experimental data indicates that cardiomyocytes, thanks to the increased expression and function of TLR4 present on their surface, become pro-inflammatory cells in hearts which are affected by post-infarction damage. The importance of increased affinity of TLR4 on CHF cardiomyocytes to heat shock proteins 60 (HSP60), which is ischaemia-induced, DAMP ligand for TLR4, is emphasised. In addition, in the case of CHF cardiomyocytes, the binding of TLR4 and HSP60 results in a greater than normal production of pro-inflammatory cytokines. This has been shown to happen up to 4 weeks after MI. In this same period, no TLR4 was found on the surface of monocytes/macrophages infiltrating the examined heart area [37]. This data strongly suggests that cardiomyocytes play an active role in initiating and sustaining inflammation in the heart after a MI.

In the animal model of diastolic HF, it was confirmed, furthermore, that persistent activation of toll-like receptor 9 (TLR9) induces systemic and cardiac inflammatory response and increases diastolic dysfunction of the heart [48]. It is now believed that stimulation of both cardiac and non-cardiac TLR9 leads to the activation of NF-kB and interferon regulatory factor 3/7 (IRF 3/7). This, in turn, results in the release of large amounts of various pro-inflammatory cytokines and chemokines. In the animal model of experimentally induced left ventricular diastolic dysfunction, it was confirmed that the degree of diastolic dysfunction was associated with the degree of myocardial inflammation, of the intensity of pro-inflammatory cytokines expression and of myocardial infiltration by monocytes/macrophages [48]. The animal model used in this experiment does not allow the effects caused by direct TLR9 stimulation to be fully differentiated from the effects resulting from systemic inflammation. It is emphasised, however, that the relationship between TLR9 stimulation and the severity of diastolic dysfunction is sufficiently proven [48].

## Sema4D

Recently, the interest of researchers has also been aroused by semaphorin 4D (Sema4D), a transmembrane glycoprotein present mainly on platelets and T lymphocytes. Sema4D is considered to be a glycoprotein involved mainly in inflammatory processes, although it may be also associated with embryonic development and angiogenesis [49]. When determining serum concentrations of Sema4D in patients with HF, significantly higher concentrations were found than in a healthy control group [49, 50]. In addition, a significant increase in the plasma concentration of Sema4D during acute exacerbation of CHF and a rapid reduction in the concentration of this parameter after clinical improvement were observed in the HF group. In the entire study group, serum concentrations of Sema4D correlated well with the clinical state of the patients, expressed by the NYHA functional class and with the plasma concentration of NT–proBNP. The increase in Sema4D concentration did not depend, however, on left ventricular ejection fraction (LVEF). A difference was shown here in relation to NT–proBNP, the concentration of which was dependent on LVEF. During observation of patients hospitalised due to the exacerbation of HF, there was a significant decrease in Sema4D serum concentration during their hospitalisation after clinical improvement. In the same group of patients, plasma NT–proBNP concentrations were not significantly reduced [49]. For this reason, Sema4D is currently considered as a potential biomarker for acute HF exacerbation, allowing for the diagnosis of acute HF and for the monitoring of the clinical course of HF.

## The role of the TGF-Beta1/Smad3 signalling pathway in inflammation in HF

Transforming growth factor-beta 1 (TGF-Beta1) is a cytokine with multidirectional action, regulating such processes as proliferation, differentiation or apoptosis of cells through autocrine and paracrine signalling pathways, involving different receptors on the cell surface. TGF-Beta1 also acts as a regulator of extracellular matrix synthesis, repair processes to damaged tissue and the functioning of the immune system [51–54]. TGF-Beta1 exerts its biological effects by binding to its receptors: TGF-Beta1 type-I receptor (TBetaRI) and type II (TBetaRII). It is believed that the above-mentioned activities of TGF-Beta1, associated with repair processes after damage to the myocardium and left ventricular remodelling, occur mainly through the TGF-Beta1/small mothers against decapentaplegic homolog 3 (Smad3) signalling pathway. The binding of TGF-Beta1 to its TBetaRI and TBetaRII receptors results in phosphorylation and the activation of Smad proteins that have the ability to bind to specific DNA and act as transcription factors. In this way, they regulate the expression of various cytokines, including, among others, platelet-derived growth factor (PDGF), fibroblast growth factor (FGF) and tumour necrosis factor (TNF). It has been shown that the inhibition of the TGF-Beta1/Smad3 signalling pathway reduces collagen synthesis in CFs, reduces the severity of myocardial fibrosis and prevents adverse remodelling in the event of pathological overload or damage to the left ventricle [55–57].

In the animal HF model, it was also confirmed that the intensity of the inflammatory process within the myocardium is significantly reduced by inhibiting the TGF-Beta1/Smad3 signalling pathway [58]. Elevated serum concentrations of pro-inflammatory cytokines such as interleukin-1Beta (IL-1Beta), IL-6 and TNF-alpha, which are typical in HF, were significantly reduced by treatment with epigallocatechin gallate (EGCG), a substance that strongly inhibits the TGF-Beta1/Smad3 signalling pathway [58]. EGCG is a catechin, i.e. a monomeric aglycone, which belongs to the group of polyphenol compounds belonging to flavonoids. EGCG is obtained mainly from leaves or buds of Camellia sinensis [58]. EGCG is also common in many foods such as apples, apricots, cocoa, black and green tea, red wine and legumes [59]. Previous studies have confirmed that EGCG effectively reduces myocardial hypertrophy and adverse left ventricular remodelling caused by pressure overload. In the animal model of pressure overload, EGCG has also been shown to prevent apoptosis of cardiomyocytes, reduce oxidative stress and inhibit abnormal proliferation of CFs [60, 61]. It has been confirmed that the inhibition by EGCG of fibroblast proliferation and excessive collagen production takes place by disrupting the functioning of the TGF-Beta1/Smad3 signalling pathway [62]. Recent animal studies confirm that EGCG significantly inhibits the inflammation in HF by disrupting the functioning of this particular signalling pathway. This correlates with the reduction in plasma concentrations of BNP and NT-proBNP, with improvement of left ventricular systolic function and of left ventricular dimensions, as well as with a survival index in this group of animals with HF [58]. The results of these studies give a preliminary theoretical basis for the treatment of the TGF-Beta1/Smad3 signalling pathway as a potential therapeutic target in HF.

## The participation of micro-RNA in inflammation in HF

Micro-RNAs (miRNAs) are a group of small, non-coding RNA molecules that regulate the expression of genes in the transcriptional and post-transcriptional stages. MiRNAs are the largest group of so-called short, regulatory RNAs, also known as small regulatory RNAs (srRNAs). MiRNAs are involved in gene silencing at post-transcriptional or transcriptional stages [63]. Genes for miRNAs occur in various locations. They can occur in introns and exons of structural genes and in intergenic regions. MicroRNAs are designated with the abbreviation miRNA or more often miR, to which the appropriate numbers, identifying the appropriate type of microRNA, are appended [63, 64].

The growing interest in miRNAs in people results from the role that these molecules play in many important physiological and pathological processes. It has been shown that miRNAs in humans are involved in, among other things, the regulation of processes such as hematopoietic stem cell differentiation, neurogenesis, embryogenesis, angiogenesis, insulin secretion, differentiation of mononuclear cells and the formation and activity of immune system cells [65–68]. Moreover, their significance in such conditions as inflammation, cancers, autoimmune diseases and cardiovascular diseases has been confirmed [64, 69–82]. The usefulness of miRNAs as biomarkers in cardiovascular diseases results from their participation in pathophysiological processes related to cardiovascular diseases as well as their stability in blood and urine [83].

Recently, particular attention has been paid to certain miRNAs because of their role in the regulation of the function of both vascular and cardiac endothelial cells and because of their effect on left ventricular remodelling after MI. More and more research is providing evidence for the key role of miRNAs in the course of MI and in post-infarction left ventricular remodelling. This was shown, inter alia, for miR-532, miR-145, miR-155, miR-27a and miR-150 [84–88]. The influence of miRNAs on the intensity of myocardial fibrosis processes after MI was also examined with regards to their influence on the expression of TGF-Beta1, which is a known mediator of organ fibrosis processes and regulates the function of fibroblasts [89]. As described above, TGF-Beta1 also regulates the severity of the inflammatory process in the myocardium during HF. In addition, the inhibition of the TGF-Beta1/Smad3 signalling pathway causes a reduction in plasma concentrations of pro-inflammatory cytokines such as IL-1Beta, IL-6 or TNF-alpha, all of which are elevated in HF. [58]. Increased expression of miR24 has been shown to inhibit the expression of TGF-Beta1 in CFs [89]. The decreased expression of miR-24 in the acute phase of MI has been associated with the intensification of cardiac fibrosis mainly in the infarction area and in the border zone of the myocardial necrosis. An inverse correlation between miR-24 expression and the amount of collagen type 1, fibronectin and TGF-Beta1 was demonstrated in different areas of the mouse heart which had undergone an experimentally induced MI [89].

Recently, miR-146a and miR-486 have aroused particular interest, mainly in the context of the contribution of inflammation to the pathogenesis of HF. They are considered to be an element of the inflammatory network, in which NF-kappaB plays a key role, by increasing the concentration of pro-inflammatory cytokines such as IL-1, IL-6, TNF-alpha and TNF-gamma. These cytokines, in turn, activate NF-kappaB, which forms a positive feedback loop. NF-kappaB increases the expression of miR-146a, which inhibits the action of the IL-1 and TNF-alpha receptors, thereby decreasing inflammation. For this reason, the cardioprotective effect of miR-146a has been highlighted recently [90]. Lately, a trend towards elevated plasma concentration of miR-146a and miR-486 has been demonstrated in a group of patients with HF when compared with a control group [90]. In addition, NF-kappaB reduces the level of muscle-specific transcription factor (MyoD). MyoD, together with myocardin-related transcription factor (MRTF), positively regulates the expression of miR-486. The balance between the activity of MRTF and MyoD in inflammatory conditions determines the level of miR-486 expression in the myocardium [90].

## Ageing as a factor in inflammation and as a promoter of the development of HF

The natural ageing process leads to structural and functional changes in the heart, which include, inter alia, inflammation and fibrosis which promote the development of HF. In studies in rats, it was shown that during normal ageing, there is a significant increase in myocardial infiltration by macrophages and in the gene expression for pro-inflammatory cytokines [91]. It has been found, for example, that the ageing process significantly activates NF-kB, a key regulator of gene transcription for pro-inflammatory factors in the myocardium. It has also been well proven in various studies that, in the ageing process, interferon gamma (INF-gamma), IL-6, lipopolysaccharide (LPS), TLR4 and TGF-Beta1 are also activated in the myocardium [91, 92]. Interestingly, ageing has also been shown to inhibit the expression of Smad7, known as a TGF-Beta1 inhibitor and myocardial fibrosis inhibitor [91]. All of the above effects of ageing have been confirmed in the previously mentioned experimental model, but only in female specimens. In this study, extremely interesting results were obtained regarding the possibility of reversing the age-induced pro-inflammatory and profibrotic processes which lead to the development of HF. It was shown that the use of relaxin (RLX), administered subcutaneously over a period of 2 weeks, leads to the suppression of INF-gamma, IL-6, LPS, TLR4 and TGF-Beta1 expression and to the activation of Smad7. This effect occurs in both male and female specimens [91]. It was also confirmed by analysis of the transcription of the atrial natriuretic peptide (ANP) gene that a significant increase in ANP expression in the left ventricular muscle of the examined female rats occurs during the ageing process. In male rats, differences relating to age were not statistically significant. However, in terms of the effect of RLX on ANP expression, a significant reduction in ANP expression was observed in both sexes after the use of RLX for 2 weeks [91]. This gives hope for the future use of the beneficial effects of RLX in the treatment of HF and other inflammatory diseases, although further research in this area is necessary.

In addition, in a group of elderly people aged 70–82 without a previous HF diagnosis, it was shown that the presence of markers of systemic inflammatory reaction, such as CRP or IL-6, was associated with a higher risk of hospitalisation for HF and a higher rate of cardiovascular mortality. These markers also correlated positively with a higher rate of resting heart rhythm [93]. The study group was separated from the PROSPER (Prospective Study of Pravastatin in the Elderly at Risk) study population, excluding those who used beta-blockers. The described increase in risk was independent of the classic cardiovascular risk factors. It is now believed that systemic inflammation can accelerate the resting rate of heart rhythm by affecting the autonomic nervous system. Accelerated resting heart rhythm is, in turn, associated with a higher cardiovascular risk and the risk of hospitalisation due to HF [93]. The mechanism of this relationship is partly explained by inflammation and partly by other mechanisms associated with endothelial dysfunction or neurohormonal activation [93, 94]. The importance of resting heart rate as a prognostic factor and, at the same time, a therapeutic goal is well-documented particularly in a group of patients with HFrEF with LVEF less than or equal to 35% [1, 94]. In this group of patients, where the sinus rhythm is present and symptoms remain, despite the classic HF treatment, the benefits of treatment with ivabradine, a specific factor inhibiting the formation of the If current in the sinus node, were demonstrated [1, 94]. It is now proposed that this beneficial effect of ivabradine may result not only from the effects on heart rate, but also from changes induced by ivabradine in the immune system. It has been demonstrated that in patients treated with ivabradine, contemporaneous with the reduction in heart rate, there is a significant reduction in plasma TNF-alpha concentration and the restoration of the correct level of circulating dendritic cells [94]. In a study in which these ivabradine-induced changes in the immune system were confirmed, these were significant in dilated cardiomyopathy (DCM) and ischaemic cardiomyopathy (ICM), but not in hypertensive cardiomyopathy (HCM). At the same time, the reduction in heart rate was identical in all these groups. It is believed that this may be an indication that some of the beneficial effects of ivabradine in HF may be independent of the impact on the heart rhythm and result from its potential additional (pleiotropic) activities [94].

## Inflammation as a target in the therapy of HF—history and future perspectives

Due to the growing amount of evidence confirming the role of inflammation in the pathogenesis of HF, attempts are being made to develop a therapeutic strategy based on the inhibition of the selected pathway of inflammation in HF. So far, however, in those clinical trials evaluating such “anti-inflammatory” therapies, no evidence has been found for their beneficial effects in patients with HF. On the other hand, there is a consensus that these studies should be continued [2, 4].

One such concept of treatment was based on the blocking of TNF-alpha activity, due to its proven pro-inflammatory effect in HF. In two clinical trials, etanercept was studied in a total population of 1500 patients with HF. This was the RECOVER study (Research into Etanercept Cytokine Antagonism in Ventricular Dysfunction) and the RENAISSANCE study (Randomised Etanercept North American Strategy to Study Antagonism of Cytokines). None of these studies demonstrated the benefits of etanercept in patients with HF, and, in the RENAISSANCE study, there was even a significant deterioration in HF [2, 4, 95]. In the ATTACH (Anti-TNF Therapy against Congestive Heart Failure) study, infliximab, which is a monoclonal antibody directed against TNF-alpha, was also investigated. In this study, mortality and hospitalisation caused by the exacerbation of HF increased in the patients who were treated with infliximab [2, 4, 96].

Also, dexamethasone treatment was no better than a placebo in patients with idiopathic DCM [97]. Statins in HF have been studied in such studies as CORONA (Crestor versus Placebo in Subjects with Heart Failure) or GISSI-HF (Effect of Rosuvastatin in Patients with Chronic Heart Failure). In these studies, there was no beneficial effect on cardiovascular mortality or the number of hospitalisations in patients with HF [98, 99]. Due to reports of a lower frequency of HF in rheumatoid arthritis (RA) patients treated with methotrexate, a small clinical trial was also conducted with this drug in the ischaemic HF group. This was the METIS (Methotrexate Therapy on the Physical Capacity of Patients with Ischaemic Heart Failure) study, in which no benefits were demonstrated for the use of methotrexate in patients with this form of HF. [100].

Ambiguous results were obtained in trials using intravenous immunoglobulins (IVIg). In some studies, the benefits of such therapy compared to a placebo were not confirmed, while in others, IVIg improved LVEF in patients with both ischaemic and non-ischaemic HF [4]. Prolonged observation of the study group showed that approximately 1 year after the discontinuation of IVIg, there was a reduction of LVEF in these patients once again. This indicates the need for long-term use of this therapy to maintain its beneficial effects [4].

On account of experimental data confirming the presence of various antibodies in the blood of patients with idiopathic DCM, clinical trials using immunoadsorption were also carried out. These were randomised studies conducted on small groups of patients with idiopathic DCM, in which different types of antibodies were eliminated from the blood, including autoantibodies against beta-1 adrenergic receptors [4, 101–104]. In these studies, it was shown that immunoadsorption results in improved left ventricular function in this group of patients. However, the observed improvement of the left ventricular function was present only in those patients with DCM where the presence of cardiodepressive antibodies had been initially confirmed [101–105].

Another study, the ACCLAIM (Advanced Chronic Heart Failure Clinical Assessment of Immune Modulation Therapy) study investigated the effect of non-specific immunomodulation on the HF process and prognosis in this group of patients. In this study, a blood sample collected from the patient was treated externally with a gaseous mixture of oxygen and ozone, and then, this blood sample was administered to the patient in the form of an intragluteal injection to induce a beneficial immune system response. In this study, no significant reduction in cardiovascular mortality or reduction in hospitalisation due to HF was achieved [106].

In animal studies, the role of pentraxin (PTX) in the development of left ventricular damage and of HF was evaluated [4]. PTX is a molecule whose expression is confirmed within vascular endothelial cells, smooth myocytes, adipocytes and fibroblasts. It has been confirmed that the production of PTX3 is stimulated by such inflammatory signals as IL-1 and TNF-alpha. Particularly, high levels of PTX3 expression in the heart and increased production of PTX3 by vascular endothelial cells were found during the activation of the inflammatory reaction. The role of PTX during the acute phase of MI was studied in an animal model of MI in PTX3 knockout mice. It was found that the lack of PTX3 results in a larger area of the lesion, a larger neutrophil infiltrate, increased apoptosis of cardiomyocytes and fewer capillaries in the myocardium during MI. In addition, the administration of exogenous PTX3 resulted in a protective effect in this group of mice [107]. For this reason, PTX3 is considered as a potential therapeutic tool, protecting against early damage of the myocardium due to MI. Further research is, however, necessary.

## Conclusions

As outlined above, there are many aspects of inflammation pathogenically associated with HF. At the same time, despite unambiguous evidence of the involvement of the immune system and pathways inducing and supporting inflammation in the pathogenesis of HF, attempts to target these pathways have not given the expected beneficial effects up to this point. This is probably due to the large variety of inflammatory pathways in different types of HF. Other inflammatory pathways are responsible for the size of myocardial ischaemic damage and post-infarction left ventricular remodelling, while others dominate in HF of non-ischaemic aetiology. Therefore, the search for a common inflammatory pathway characterising all forms of HF seems inappropriate from the point of view of building a concept of treatment that inhibits the inflammatory process. A good example is the efficacy of immunoadsorption therapy in a selected group of patients with idiopathic DCM, in whom the presence of cardiodepressive antibodies has been confirmed. In the entire population of patients with DCM, this therapy is ineffective. However, when precise selection and appropriate qualification of patients for immunoadsorption therapy is made, a significant improvement in the left ventricular function is obtained. For this reason, further research is needed to understand the complex pathophysiological mechanisms involving the various inflammatory pathways in different types of HF. A better understanding of them will allow the identification of specific subsets of HF patients for whom specific anti-inflammatory treatment can be tailored. Considering how little we understand these complex pathophysiological mechanisms currently, we still have a long way to go to create good, tailor-made anti-inflammatory therapies that effectively improve the prognosis in HF.
